# Toxicity of 6:2 Chlorinated Polyfluorinated Ether Sulfonate (F-53B) to *Escherichia coli*: Growth Inhibition, Morphological Disruption, Oxidative Stress, and DNA Damage

**DOI:** 10.3390/microorganisms13122819

**Published:** 2025-12-11

**Authors:** Jun Di, Zinian Li, Lixia Yuan, Jinxian Liu, Baofeng Chai

**Affiliations:** 1 Department of Biological Science and Technology, Jinzhong University, Jinzhong 030619, China; dijun1888@163.com (J.D.); leazinian@163.com (Z.L.); lixiayuan1999@126.com (L.Y.); 2 Key Laboratory of Shanxi Province for Ecological Restoration of Loess Plateau, Institute of Loess Plateau, Shanxi University, Taiyuan 030006, China; liujinxian@sxu.edu.cn

**Keywords:** F-53B, *Escherichia coli*, toxicity mechanism, oxidative stress, DNA damage

## Abstract

6:2 chlorinated polyfluoroalkyl ether sulfonic acid (F-53B), a substitute for perfluorooctane sulfonate (PFOS), is widely used as a mist suppressant in the electroplating industry. With the implementation of PFOS regulations, the use of F-53B has correspondingly increased, and it is now detected in various environmental matrices. However, toxicological information on F-53B remains incomplete and insufficient for environmental risk assessment. In this study, we systematically investigated, for the first time, the toxicity and underlying mechanisms of action of F-53B to *Escherichia coli*. The results showed that the 24 h half-maximal growth inhibition concentration (IC_50_) of F-53B was 23.56 mg/L, suggesting that F-53B may exhibit higher toxicity to *E. coli* than PFOS. Analyses of cell surface hydrophobicity, membrane permeability, membrane composition, and scanning electron microscopy (SEM) images showed that F-53B adsorbed onto the cell surface, altered membrane properties, and ultimately disrupted cell morphology. Increased intracellular levels of reactive oxygen species (ROS) and malondialdehyde (MDA), along with decreased activities of superoxide dismutase (SOD) and catalase (CAT), indicated enhanced oxidative stress induced by F-53B in *E. coli*. Furthermore, the alkaline comet assay demonstrated that F-53B exposure caused DNA damage. Taken together, the toxicity of F-53B to *E. coli* can be attributed to cell morphological disruption, oxidative stress, and DNA damage, ultimately leading to cellular inactivation or death. These findings advance our understanding of the cytotoxicity of F-53B in microorganisms.

## 1. Introduction

Perfluorooctane sulfonate (PFOS) is one of the most chemically stable per- and polyfluoroalkyl substances (PFASs) and has been widely used in industrial and manufacturing applications since the 1940s [1]. Increasing evidence has demonstrated that PFOS is environmentally persistent, bioaccumulative, and toxic to wildlife and humans [2]. In recognition of these risks, PFOS and related substances were listed under Annex B of the Stockholm Convention on Persistent Organic Pollutants (POPs) in 2009, restricting their production and use. Since then, a variety of alternatives have emerged and drawn widespread attention. As an important PFOS alternative, 6:2 chlorinated polyfluorinated ether sulfonate (6:2 Cl-PFESA, trade name F-53B) has been extensively used as an industrial chrome mist suppressant in China, with an annual production of 30–40 t [3]. The increasingly widespread application and unregulated emissions of F-53B have resulted in its release into the environment, particularly in aquatic systems [4,5,6]. The environmental occurrence of F-53B has recently been reported in industrial wastewater and river water at concentrations up to 112 μg/L and 7.6 μg/L, respectively [7,8]. EUSES calculations predicted that F-53B concentrations in freshwater systems in South China could approach 0.7 mg/L in 2015 and potentially exceed 2.3 mg/L near chromium-plating plants by 2020 [9]. Notably, F-53B exhibits a longer biological half-life than PFOS (up to 15.3 years) and long-range migration capacity [1,10]. These features underscore the need for systematic research on the toxicological effects and mechanisms of F-53B in aquatic ecosystems.

Previous studies have reported diverse adverse effects of F-53B in fish and mammals. In adult zebrafish (*Brachydanio rerio*), F-53B exhibited moderate acute toxicity with a 96 h LC_50_ of 24.7 mg/L [8] and caused reproductive toxicity [11], embryotoxicity [12], eye development disorders [13], hepatotoxicity [14], and thyroid endocrine disruption [15]. Oxidative stress is increasingly implicated as a key mechanism underlying F-53B-induced developmental toxicity in fish [8,16]. In mammals, F-53B has been associated with subchronic reproductive toxicity [17], neurotoxicity [18], nephrotoxicity [19], vascular toxicity [20], and osteoporosis [21]. Notably, F-53B also showed significantly higher acute toxicity than PFOS to earthworms (*Eisenia fetida*), with a 7 d LC_50_ of 1.43 mmol/kg dry soil [22].

Bacteria are ubiquitous in aquatic ecosystems and play crucial roles in microbial food webs and ecosystem functioning [23]. In addition, as single-cell organisms, bacteria serve as important models for evaluating ecotoxicity and investigating underlying mechanisms at the cellular level. Research has indicated that exposure to F-53B can cause significant shifts in bacterial community structure and diversity, leading to impaired nitrification functions [24]. However, the mechanisms underlying the effects of F-53B on bacteria remain unclear. The bacterial toxicity of PFASs has been investigated in only a few studies. For instance, Nobels et al. [25] employed a multiple-endpoint bacterial reporter assay to characterize the toxicological profiles of PFASs. Another study demonstrated that exposure to PFASs increases membrane permeability and induces a quorum-sensing response in *Aliivibrio fischeri* [26]. Furthermore, the bacterial toxicity of PFOS has been more extensively studied and is associated with membrane disruption, oxidative stress, and genetic damage [26,27,28]. Given the structural similarity between PFOS and F-53B, F-53B may exert comparable biotoxic effects. Nevertheless, information regarding the toxicity of F-53B to microorganisms and its underlying mechanisms has not yet been reported.

This study aimed to evaluate the toxicity and mechanisms of action of F-53B to bacteria. *Escherichia coli* was selected as the target organism due to its short growth cycle, ease of manipulation, and well-defined genetic background. Moreover, *E. coli*, as a key indicator of contamination and health risks in environmental waters, has been widely used for ecological risk assessment [28,29,30]. Therefore, the results of this study will help elucidate the toxicity mechanisms of F-53B at the cellular level and provide a basis for assessing its ecological risks.

## 2. Materials and Methods

### 2.1. Strain, Chemicals, and Reagents

*E. coli* (ATCC 9080) was obtained from the China General Microbiological Culture Collection Center (Beijing, China). F-53B (CAS no. 73606-19-6; purity ≥ 99%) was purchased from Maikun Chemical Co., Ltd. (Shanghai, China). A 1 g/L stock solution of F-53B was prepared in a mixture of dimethyl sulfoxide (DMSO) and ultrapure water for exposure experiments. Prior to use, the stock solution was filter-sterilized through a 0.22 μm membrane filter. The final concentration of DMSO in the assay medium was maintained below 0.6% (*v*/*v*), at which no significant effect on the growth of *E. coli* was observed. Assay kits for 4′, 6-diamidino-2-phenylindole dihydrochloride (DAPI), reactive oxygen species (ROS), superoxide dismutase (SOD), catalase (CAT), and malondialdehyde (MDA) were obtained from Beijing Solarbio Science & Technology Co., Ltd. (Beijing, China). All other chemicals were purchased from Beyotime Biotechnology (Shanghai, China).

### 2.2. Bacterial Toxicity Assessment

The exposure experiment followed a previously described method [27] with minor modifications. *E. coli* was cultured overnight at 37 °C with shaking (160 rpm) in BHI broth medium (proteose peptone 10 g/L, Na_2_HPO_4_·12H_2_O 2.5 g/L, beef heart infusion 17.5 g/L, NaCl 5 g/L, and dextrose 2 g/L; pH 7.4 ± 0.2) to obtain sufficient inoculum. Bacterial cultures in the exponential phase (OD_600_ = 0.3) were exposed to different concentrations of F-53B (0, 0.1, 1, 10, 50, 100, 200, and 300 mg/L). All cultures were incubated at 35 °C without shaking. Samples were collected at 0, 2, 4, 8, 12, 16, 20, 24, and 28 h post-exposure, respectively. Optical density at 600 nm (OD_600_) was measured using a microplate reader (Infinite^®^ 200 Pro, TECAN, Männedorf, Switzerland) to generate growth curves. The growth inhibition rate was calculated using Equation (1) [31]. The 24 h IC_50_ was determined by non-linear regression analysis.

Inhibition rate (%) = [1 − (OD_600_ of treated cells/OD_600_ of control cells)] × 100 (1)

### 2.3. SEM Analysis

*E. coli* morphology and membrane integrity were examined by scanning electron microscopy (SEM) according to a previously described method [27]. After exposure to 0, 0.1, 10, and 50 mg/L F-53B for 24 h, cells were harvested by centrifugation (8000 rpm, 5 min), fixed with 2.5% glutaraldehyde, and washed three times with 0.2 M PBS (pH 7.0). Samples were gradually dehydrated with graded ethanol (30%, 50%, 70%, 85%, 95%, and 100%; 20 min each), treated with tert-butanol, dried in a CO_2_ critical-point desiccator, and coated with a thin layer of gold-palladium. Cell morphology was observed using an SEM (SU5000, Hitachi, Tokyo, Japan).

### 2.4. Measurement of Cell Surface Hydrophobicity

Cell surface hydrophobicity (CSH) was determined using the microbial adhesion to hydrocarbons (MATH) assay according to a recorded method [28]. After exposure to 0, 0.1, 10, and 50 mg/L F-53B for 24 h, *E. coli* cells were collected by centrifugation (4000 rpm, 5 min, 4 °C), washed twice with 0.9% NaCl (5000 rpm, 10 min), and resuspended in 0.9% NaCl to OD_600_ ≈ 1.0 (A_0_). Two milliliters of the suspension were mixed with 2 mL xylene, vortexed vigorously for 2 min, and allowed to phase-separate at room temperature for 30 min. The OD_600_ of the aqueous phase was measured and recorded as A. CSH (%) was calculated using Equation (2) [28].

CSH (%) = [1 − (A/A_0_)] × 100 (2)

### 2.5. Measurement of Membrane Permeability

Membrane permeability was evaluated using DAPI, a membrane-permeable DNA-binding dye whose fluorescence increases upon binding to DNA [27]. After 24 h exposure to 0, 0.1, 10, and 50 mg/L F-53B, cells were harvested by centrifugation (4000 rpm, 10 min, 4 °C), washed three times with 0.2 M PBS (pH 7.0), and resuspended in PBS containing DAPI (4 μg/mL). After 10 min incubation, fluorescence was measured (excitation/emission: 355/460 nm) and recorded as A. Cells were then lysed through five freeze–thaw cycles to release total DNA, incubated with DAPI for 10 min, and the maximum fluorescence was measured (excitation/emission: 355/460 nm) and recorded as A_0_. Membrane permeability (expressed as dye leakiness) was calculated using Equation (3) [27].

Dye leakiness (%) = (A/A_0_) × 100 (3)

### 2.6. ATR-FTIR Analysis

Attenuated total reflectance Fourier transform infrared spectroscopy (ATR-FTIR; Nicolet iS50, Thermo Fisher Scientific, Waltham, MA, USA) was used to analyze changes in cellular chemical composition according to a previously described method [32]. After 24 h exposure to 0, 0.1, 10, and 50 mg/L F-53B, cells were washed with 0.2 M PBS (pH 7.0) and freeze-dried to remove residual water. ATR-FTIR spectra were recorded in the range of 4000–500 cm^−1^ with 32 scans at a resolution of 4 cm^−1^. The spectra were processed using OMNIC 9.2 software with baseline correction.

### 2.7. Determination of Oxidative Stress

Intracellular ROS levels were determined using the fluorescent probe 2′,7′-dichlorofluorescein diacetate (DCFH-DA) after 24 h exposure to 0, 0.1, 10, and 50 mg/L F-53B, following a previously described method [28] with minor modifications. Cells were collected by centrifugation (4000 rpm, 5 min, 4 °C), washed three times with 0.2 M PBS (pH 7.0), resuspended in PBS, and incubated with 20 µM DCFH-DA at 37 °C for 60 min in the dark. After incubation, cells were washed three times with 0.2 M PBS (pH 7.0) and resuspended to approximately 1 × 10^6^ cells/mL. Fluorescence intensity (FI) was measured at excitation and emission wavelengths of 488 nm and 525 nm, respectively. Relative ROS levels were calculated using Equation (4) [33].

Relative ROS levels = FI of treated cells/FI of control cells(4)

For antioxidant and lipid peroxidation biomarkers, cells were harvested by centrifugation (6000 rpm, 10 min), washed three times with 0.2 M PBS (pH 7.0), and resuspended to approximately 5 × 10^6^ cells/mL. Cell lysis was performed on ice using an ultrasonic cell disruptor (Scientz Biotechnology Co., Ltd., Ningbo, China; 200 W) for 30 cycles (3 s pulse, 10 s rest). Homogenates were centrifuged (8000 rpm, 10 min, 4 °C) and supernatants were collected for biochemical analysis. SOD and CAT activities and MDA content were measured using commercial assay kits in accordance with the manufacturer’s instructions, as described in the Table S1.

### 2.8. Alkaline Comet Assay

The alkaline comet assay was performed according to a method [29], with minor modifications. After 24 h exposure, cells were harvested by centrifugation (1500 rpm, 10 min), washed three times with 0.2 M PBS (pH 7.4), and resuspended to approximately 1 × 10^5^ cells/mL. A 50 µL aliquot of the cell suspension was incubated with lysozyme (1 mg/mL) and RNase A (100 μg/mL) at 37 °C for 30 min, mixed with 500 µL of 0.7% low-melting agarose, and immediately spread onto microscope slides pre-coated with a layer of 1% normal-melting agarose. After solidification, slides were immersed in lysis solution (2.5 M NaCl, 100 mM Na_2_EDTA, 10 mM Tris-HCl, and 1% Triton X-100; pH 10) overnight at 4 °C in the dark. Slides were then subjected to alkaline unwinding in an electrophoresis buffer (300 mM NaOH, 1.2 mM Na_2_EDTA) for 30 min and electrophoresed at 25 V (300 mA) for 20 min. After neutralization with 0.4 mM Tris-HCl (pH 7.5), slides were air-dried and stained with SYBR Green I. Comets were observed using an inverted fluorescence microscope (Axio Observer D1, Zeiss, Oberkochen, Germany) and analyzed with CASP 1.2.3 software. Tail DNA% (TDNA) and Olive tail moment (OTM) were recorded. The extent of DNA damage was further classified based on TDNA as follows [34]: <5% (no damage), 5–20% (mild damage), 21–40% (moderate damage), 41–95% (high damage), and >96% (severe damage).

### 2.9. Statistical Analyses

Experiments were performed in triplicate, and data are presented as means ± standard deviation (SD). Statistical analyses were conducted using SPSS 29.0. Significant differences were analyzed by one-way ANOVA followed by Dunnett’s test or the least significant difference (LSD) test. Differences were regarded as significant at *p* < 0.05.

## 3. Results

### 3.1. Effect of F-53B on the Growth Profile

**Figure 1:**
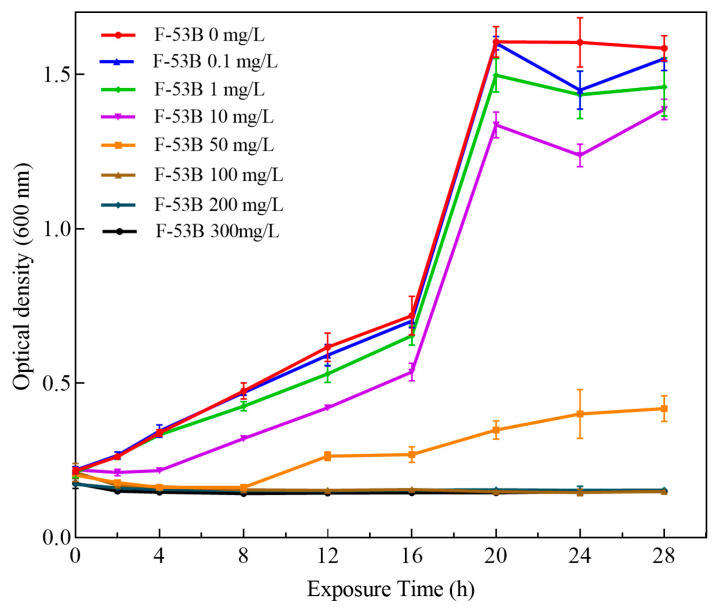
Growth curve of *Escherichia coli* exposed to different concentrations of F-53B for 28 h. Error bars represent standard deviations (*n* = 3).

**Figure 2:**
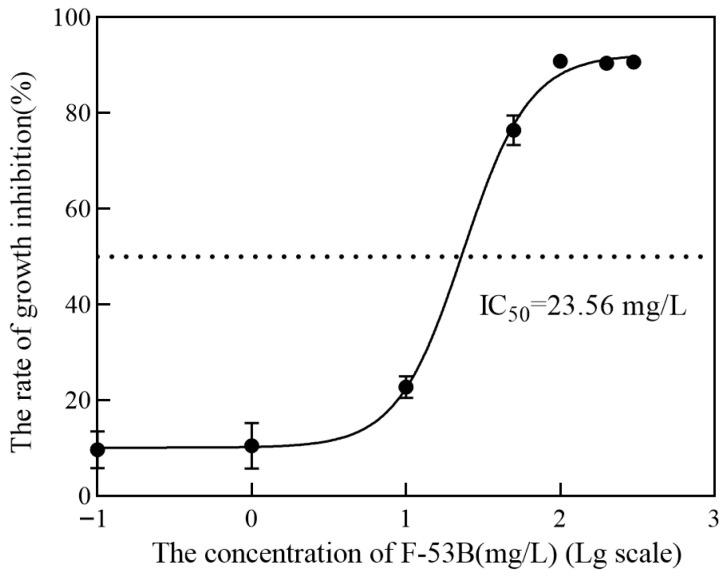
Dose–response curve of *Escherichia coli* after 24 h of exposure to F-53B at concentrations of 0, 0.1, 1, 10, 50, 100, 200, and 300 mg/L. Error bars represent standard deviations (*n* = 3).

Bacterial toxicity was evaluated by analyzing the growth curve and growth inhibition rate of *E. coli* exposed to F-53B at concentrations of 0, 0.1, 1, 10, 50, 100, 200, and 300 mg/L. The growth curve was monitored by measuring OD_600_ at predetermined time intervals until the stationary phase. During the entire exposure period, F-53B exhibited dose-dependent growth inhibition at concentrations ranging from 0.1 to 300 mg/L (Figure 1). After 20 h, strong inhibition of *E. coli* growth (up to 90%) was observed at concentrations ≥ 100 mg/L. These results indicate that high doses of F-53B suppress *E.coli* cell division. The 24h IC_50_ for *E. coli* was determined to be 23.56 mg/L, with a 95% confidence interval of 20.49–27.05 mg/L (Figure 2).

### 3.2. Effect of F-53B on Cellular Morphology

**Figure 3:**
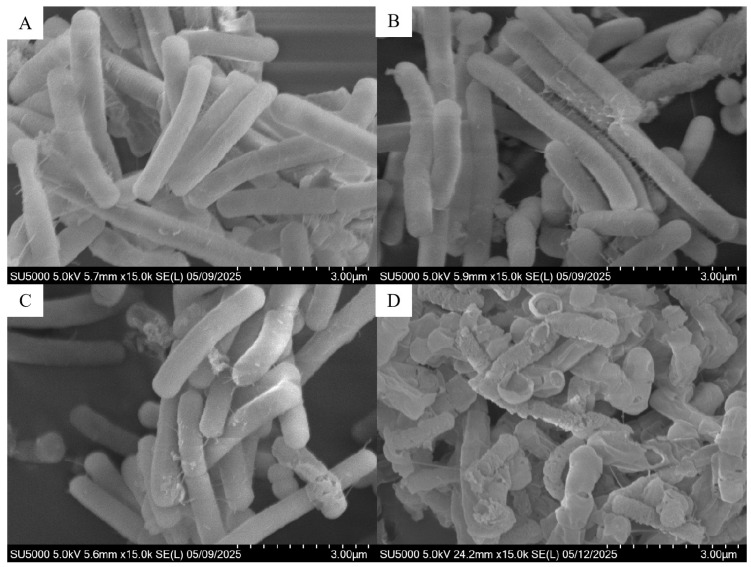
Scanning electron microscopy (SEM) images of *Escherichia coli* after 24 h of exposure to F-53B: 0 mg/L (**A**), 0.1 mg/L (**B**), 10 mg/L (**C**), and 50 mg/L (**D**).

SEM images were used to reveal morphological changes in *E. coli* induced by F-53B (0, 0.1, 10, and 50 mg/L). Untreated cells displayed intact, rod-shaped morphology with a smooth surface (Figure 3A). At 0.1 mg/L F-53B, cellular morphology remained largely unchanged, indicating negligible effects on cell integrity at this low concentration (Figure 3B). When the concentration increased to 10 mg/L, noticeable disruptions appeared in a subset of cells (Figure 3C). At the highest concentration (50 mg/L), *E. coli* exhibited severe cellular damage, characterized by prevalent cell lysis and cytoplasmic leakage (Figure 3D). These findings demonstrate that F-53B induces concentration-dependent morphological changes in *E. coli*, with pronounced cellular damage occurring at higher concentrations.

### 3.3. Effect of F-53B on Cell Surface Properties

**Figure 4:**
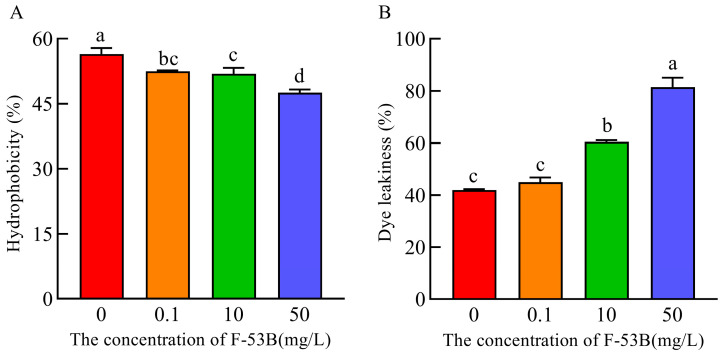
Cell surface hydrophobicity (**A**) and membrane permeability (**B**) of *Escherichia coli* after 24 h of exposure to different concentrations of F-53B (0, 0.1, 10, and 50 mg/L). Values with different letters (a–d) indicate statistically significant differences (*p* < 0.05). Error bars represent standard deviations (*n* = 3).

CSH of *E. coli* following 24 h of exposure to F-53B (0, 0.1, 10, and 50 mg/L) is shown in Figure 4A. CSH gradually decreased with increasing F-53B concentration. All F-53B-treated groups showed significantly lower CSH values (*p* < 0.05) than the control, indicating that even low-level exposure reduced CSH. Moreover, the 50 mg/L treatment group exhibited a further significant decrease (*p* < 0.05) compared with the 0.1 and 10 mg/L groups, whereas no significant difference was observed between the 0.1 and 10 mg/L groups. These results demonstrate a concentration-dependent decline in CSH, with the most pronounced reduction occurring at the highest F-53B level.

Membrane permeability of *E. coli* following 24 h of exposure to F-53B (0, 0.1, 10, and 50 mg/L) is shown in Figure 4B. Compared with the control, membrane permeability in the F-53B-exposed groups increased in a concentration-dependent manner. In particular, membrane permeability in the 10 and 50 mg/L F-53B treatment groups was significantly higher (*p* < 0.05) than that of the control, whereas no significant difference was observed between the control and the 0.1 mg/L group. These results indicate that marked disruption of membrane integrity occurs mainly at higher F-53B concentrations.

### 3.4. Effect of F-53B on Chemical Structures and Functional Groups

**Figure 5:**
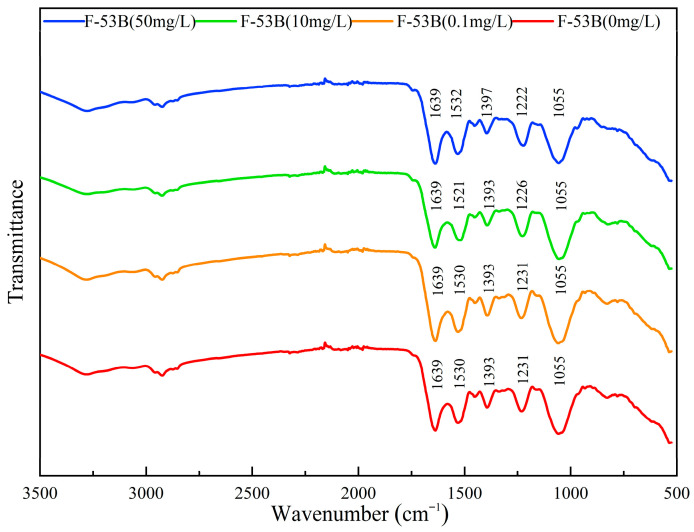
Attenuated total reflectance Fourier transform infrared spectroscopy (ATR-FTIR) of *Escherichia coli* after 24 h of exposure to different concentrations of F-53B (0, 0.1, 10, and 50 mg/L).

ATR-FTIR spectra of *E. coli* following 24 h of exposure to F-53B (0, 0.1, 10, and 50 mg/L) are shown in Figure 5. Compared with the control, cells exposed to high concentrations of F-53B (10 and 50 mg/L) exhibited red shifts in the nucleic acids region (1250–1080 cm^−1^) [35], with the characteristic peak at 1231 cm^−1^ shifting to 1226 cm^−1^or 1222 cm^−1^. This indicates that high concentrations of F-53B affect the structure of nucleic acids. In the fatty acid region (1480–1340 cm^−1^) [36], a peak shift at 1393 cm^−1^ was observed at 50 mg/L F-53B, suggesting conformational or structural changes in cellular fatty acids. Furthermore, shifts in the amide I and amide II regions (1760–1480 cm^−1^) of proteins [36] were observed at 1530 cm^−1^ following exposure to F-53B (10 and 50 mg/L), indicating structural changes in proteins at high F-53B concentrations.

### 3.5. Effect of F-53B on Oxidative Stress

**Figure 6:**
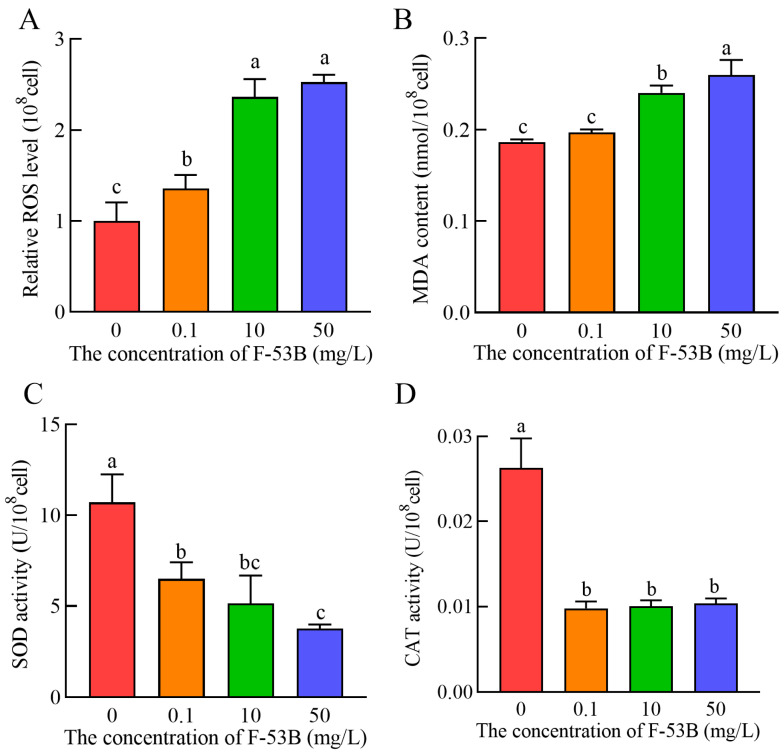
ROS levels (**A**), MDA content (**B**), SOD activity (**C**), and CAT activity (**D**) of *Escherichia coli* after 24 h of exposure to different concentrations of F-53B (0, 0.1, 10, and 50 mg/L). Values with different letters (a–c) indicate statistically significant differences (*p* < 0.05). Error bars represent standard deviations (*n* = 3).

The potential of F-53B to induce oxidative stress in *E. coli* was evaluated by measuring intracellular levels of ROS, SOD, CAT, and MDA. Following F-53B exposure, ROS levels increased in a concentration-dependent manner, with all treatment groups (0.1, 10, and 50 mg/L) exhibiting significantly higher values (*p* < 0.05) than the control, while no significant difference was observed between the 10 and 50 mg/L groups (Figure 6A). Consistently, MDA content also rose with increasing F-53B concentration, with significant elevations (*p* < 0.05) detected in the 10 and 50 mg/L groups compared with the control, whereas the 0.1 mg/L group did not differ significantly from the control (Figure 6B). In contrast, SOD activity decreased in a concentration-dependent manner, and all F-53B-treated groups exhibited significantly reduced activity (*p* < 0.05) relative to the control, with no significant difference between the 10 and 50 mg/L groups (Figure 6C). Similarly, CAT activity was also significantly suppressed (*p* < 0.05) in all treatment groups compared with the control, with no significant difference observed among the treatment groups (Figure 6D). Overall, these results indicate that F-53B triggers oxidative stress in *E. coli*, characterized by enhanced ROS and lipid peroxidation accompanied by diminished antioxidant enzyme activities.

### 3.6. DNA Damage Induced by F-53B

**Figure 7:**
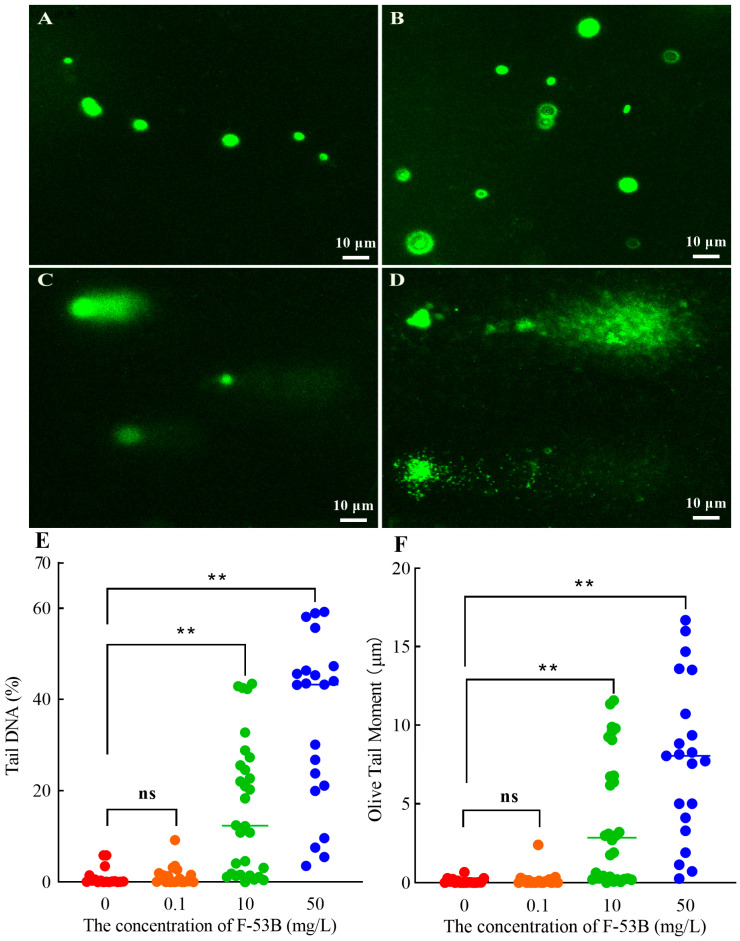
Comet assay of *Escherichia coli* exposed to F-53B for 24 h. (**A**–**D**) Representative comet assay images of cells exposed to 0, 0.1, 10, and 50 mg/L, respectively; (**E**) Tail DNA% and (**F**) Olive tail moment. Middle bars represent medians. ** *p*  <  0.01; ns, not significant.

The alkaline comet assay revealed DNA damage in *E. coli* following 24 h of exposure to F-53B (0, 0.1, 10, and 50 mg/L). Cells in the control and 0.1 mg/L F-53B groups remained intact without observed comet tails, indicating negligible DNA damage (Figure 7A,B). In contrast, higher F-53B levels led to concentration-dependent increases in comet tail length at 10 and 50 mg/L, reflecting pronounced DNA fragmentation (Figure 7C,D). Consistently, quantitative analysis showed markedly elevated TDNA and OTM values (*p* < 0.01) in these higher-concentration groups compared with the control, while no significant difference was detected between the control and 0.1 mg/L groups (Figure 7E,F). As shown in Table S1, this corresponds to mild DNA damage at 10 mg/L and moderate DNA damage at 50 mg/L, demonstrating a clear concentration-dependent genotoxic effect of F-53B.

## 4. Discussion

Ecological risk assessment of contaminants requires the evaluation of toxicity across different trophic levels. As a model prokaryotic microorganism, *E. coli* was used to assess the bacterial toxicity of F-53B. In this study, we found that, with increasing F-53B concentrations exceeding the tolerance threshold of *E. coli*, significant growth inhibition occurred. The 4 h and 24 h IC_50_ values of F-53B were 73.22 mg/L and 23.56 mg/L, respectively. Notably, these values are substantially lower than the reported 3 h IC_50_ of PFOS (374 mg/L) in *E. coli* [28], indicating that F-53B may be more toxic than PFOS in this model organism. This is consistent with the higher toxicity of F-53B relative to PFOS reported in earthworms [22], adult zebrafish [7], human hepatic cells (HL-7702) [37], and human hepatocellular carcinoma cells (HepG2 and Hep3B) [38]. These differences may be related to the higher bioconcentration factor of F-53B compared with PFOS, potentially enhancing toxicity in exposed organisms [39,40]. Furthermore, the pronounced growth inhibition of *E. coli* observed in this study suggests that the cellular-level toxicity of F-53B may contribute to its potential to disrupt microbial diversity at the community level [24]. Field monitoring in the Fen River of China revealed that F-53B was detected at all 68 sampling sites, underscoring its widespread occurrence and environmental persistence [6]. Collectively, these findings suggest that the ecological risks of F-53B as a PFOS alternative warrant serious attention.

In this study, DMSO was used as the solvent to dissolve F-53B. To minimize potential solvent effects, the final DMSO concentration in the assay medium was standardized at 0.1% (*v*/*v*) in both the control and all exposure groups during the assessment of the mechanisms of action of F-53B to *E. coli*. Previous work has demonstrated that the minimum inhibitory concentration (MIC) of DMSO for *E. coli* is approximately 15% (*v*/*v*), and that 5% (*v*/*v*) has little effect on bacterial growth [41]. Wang et al. [42] further reported that DMSO is toxic to microorganisms only at relatively high concentrations and recommended that its concentration should be kept below 2% (*v*/*v*) when used as a solvent. Vertebrate studies also indicate that DMSO concentrations up to 1% (*v*/*v*) can be safely used in zebrafish embryo developmental toxicity assays [43]. Taken together, these data indicate that the 0.1% (*v*/*v*) DMSO used in the present study is well below thresholds associated with biological interference; therefore, the effects observed can be attributed to F-53B rather than to the solvent.

To further explore the mechanism underlying F-53B-induced growth inhibition, we characterized cellular injury patterns in *E. coli*. CSH modulates interactions between bacterial cells and environmental pollutants by influencing interfacial adhesion dynamics [27]. Our study demonstrated that F-53B exposure significantly reduced CSH, consistent with reports of CSH reduction after PFOS or perfluorooctanoic acid (PFOA) exposure in *E. coli* [28]. This hydrophobicity decrease may be attributed to the amphiphilic properties of PFASs, whereby hydrophobic fluorinated tails accumulate on cell surfaces and occupy hydrophobic sites, while hydrophilic head groups orient toward the aqueous phase, thereby reducing CSH [44]. In model membranes, PFASs accumulation alters phospholipid arrangement and membrane fluidity [26]. These structural changes could be responsible for the changes in membrane permeability observed in the present study. The increase in membrane permeability can compromise cellular integrity, as evidenced by cell lysis and cytoplasmic leakage observed in the SEM images. Furthermore, PFASs have been shown to increase membrane permeability to protons, leading to diminished ATPase and metabolic activities [26,45]. Therefore, at high concentrations (10 and 50 mg/L), F-53B is likely to bind to the membrane lipid bilayer, disrupt membrane integrity, and contribute to *E. coli* inactivation or death.

ATR-FTIR is highly sensitive to subtle alterations in molecular bonding configurations, enabling discrimination of structural and biochemical perturbations within *E. coli* exposed to F-53B. In this study, ATR-FTIR analysis revealed that F-53B exposure altered the structures or compositions of proteins, fatty acids, and nucleic acids in *E. coli*. Structurally, *E. coli* possesses an outer membrane composed primarily of porins and lipopolysaccharides, with a peptidoglycan layer located between the outer membrane and the cytoplasmic membrane. The observed ATR-FTIR changes in proteins and fatty acids suggest that F-53B may partition into cellular membranes and alter cell envelope properties. This mechanism aligns with documented interactions of PFASs with phospholipid bilayers, where they disrupt acyl-chain packing and alter membrane function [46]. The alterations in nucleic acid ATR-FTIR signatures at high concentrations of F-53B (10 and 50 mg/L) indicate potential DNA damage, as further discussed below.

Oxidative stress represents a primary mechanism by which contaminants induce toxicity. Excessive ROS production is a key indicator of oxidative stress. SOD and CAT are critical antioxidant enzymes that protect against contaminant-induced ROS by detoxifying superoxide anions (O_2_^−•^) and decomposing hydrogen peroxide (H_2_O_2_), respectively [47]. Cells can enhance antioxidant defenses through activation of these antioxidant enzymes, but oxidative damage occurs when the SOD or CAT systems fail to eliminate excess ROS, leading to reduced enzyme activities and potential degradation [48]. In this study, F-53B exposure significantly decreased SOD and CAT activities, indicating disruption of the antioxidant defense system in *E. coli*. These findings are consistent with reports that F-53B impairs the antioxidant defense system in Chinese rare minnow (*Gobiocypris rarus*) [48] and zebrafish [49]. In addition, accumulated ROS can induce cell membrane lipid peroxidation, with MDA serving as an end product of lipid peroxidation and a key oxidative stress biomarker [49]. Elevated MDA content was largely consistent with increased ROS after F-53B exposure, suggesting significantly increased oxidative stress in *E. coli* and further indicating compromised cell membrane integrity. Moreover, elevated oxidative stress may induce oxidative DNA damage, leading to cellular inactivation or death [50]. Consequently, oxidative stress may serve as a sensitive biomarker for assessing F-53B toxicity risks.

The alkaline comet assay has been used to assess DNA damage in organisms following exposure to PFASs. In vivo studies have generally reported moderate genotoxic effects induced by PFASs exposure [51]. For instance, significant genotoxicity has been observed in green mussels (*Perna niridis*) [52], earthworms [53], common carp (*Cyprinus carpio*) [54], and rat (*Rattus norvegicus*) [55]. It is noteworthy that existing research has predominantly focused on eukaryotes exposed to PFOS or PFOA, whereas investigations into the genotoxic effects of their alternatives on prokaryotes remain limited. Therefore, this study investigated the genotoxic potential of F-53B to bacterial cells using the alkaline comet assay. Our results demonstrated that exposure to 10 mg/L induced mild DNA damage, while 50 mg/L exposure caused moderate DNA damage. These findings are consistent with those of Qiu et al. [56], who observed DNA damage in mouse embryonic stem cells following F-53B exposure. Previous studies have concluded that PFASs are not directly genotoxic because their chemical structures lack typical genotoxic functional groups [57]. Meanwhile, extensive evidence indicates that ROS are a major cause of DNA damage, inducing lesions through mechanisms such as strand breakage, nucleotide excision, and base modifications [53,58]. Therefore, the genotoxic effects (DNA damage) observed in this study are likely attributable to indirect mechanisms mediated by oxidative stress, whereby intracellular ROS accumulation leads to DNA damage.

One limitation of the present study is that it evaluated the toxic effects of F-53B exposure on *E. coli* only after 24 h. Further studies should assess the toxicity of F-53B over different exposure durations, as well as its impact on *E. coli* at various growth stages.

## 5. Conclusions

This study systematically investigated the toxicity of F-53B to *E. coli* and elucidated its underlying mechanisms of action. The results showed that the 24 h IC_50_ value of F-53B was 23.56 mg/L, suggesting that F-53B may exhibit higher toxicity to *E. coli* than PFOS. The toxicity mechanisms of F-53B were attributed to cell morphological disruption, oxidative stress, and DNA damage, ultimately leading to cellular inactivation or death. The results of this study advance our understanding of F-53B cytotoxicity in microorganisms and provide mechanistic evidence to support ecological risk assessment of this emerging contaminant.

## Data Availability

The original contributions presented in this study are included in the article/Supplementary Materials. Further inquiries can be directed to the corresponding author.

## Supplementary Materials

The following supporting information can be downloaded at: https://www.mdpi.com/article/10.3390/microorganisms13122819/s1; Table S1: Tail DNA% and Olive tail moment obtained from comet assay and the corresponding classification of damage type. References [59,60,61] are cited in the supplementary materials.

## Abbreviations

The following abbreviations are used in this manuscript:

F-53B	6:2 chlorinated polyfluorinated ether sulfonate
PFOS	Perfluorooctane sulfonate
IC_50_	The half maximal growth inhibition concentration
ROS	Reactive oxygen species
MDA	Malondialdehyde
SOD	Superoxide dismutase
CAT	Catalase
PFASs	Per- and polyfluoroalkyl substances
POPs	Persistent organic pollutants
PFOA	Perfluorooctanoic acid
DMSO	dimethyl sulfoxide
OD_600_	Optical density at 600 nm
SEM	Scanning electron microscope
CSH	Cell surface hydrophobicity
MATH	Microbial adhesion to hydrocarbons
ART-FTIR	Attenuated total reflectance Fourier transform infrared spectroscopy
DCFH-DA	Fluorescent probe 2′, 7′-dichlorofluorescein diacetate
FI	Fluorescent intensity
TDNA	Tail DNA%
OTM	Olive tail moment
MIC	The minimum inhibitory concentration
(O_2_^−•^)	Superoxide anions
H_2_O_2_	Hydrogen peroxide
