# Supplementary material for: Toxicity of 6:2 Chlorinated Polyfluorinated Ether Sulfonate (F-53B) to Escherichia coli: Growth Inhibition, Morphological Disruption, Oxidative Stress, and DNA Damage
Source: Microorganisms. 2025 Dec 11;13(12):2819. doi: 10.3390/microorganisms13122819 (PMC12736055; doi:10.3390/microorganisms13122819)
Supplement: Supplementary file 1 [file microorganisms-13-02819-s001.zip › microorganisms-3984804-supplementary.pdf]

Supplementary Material

Toxicity of 6:2 chlorinated polyfluorinated ether sulfonate (F-53B) to *Escherichia coli*: Growth inhibition, morphological disruption, oxidative stress, and DNA damage

Jun Dia,b, Zinian Lia, Lixia Yuana, Jinxian Liub, Baofeng Chaib*

[a]*Department of Biological Science and Technology, Jinzhong University, Shanxi, 030619, China*

[b]*Key Laboratory of Shanxi Province for Ecological Restoration of Loess Plateau, Institute of Loess Plateau, Shanxi University, Taiyuan, 030006, China*

*Corresponding author: Tel: +86-0351-7010700

E-mail: bfchai@sxu.edu.cn

Determination of superoxide dismutase (SOD) activity

SOD activity was measured according to the manufacturer's instructions, and the procedure was as follows: the supernatants were sequentially mixed with the four reagents provided in the assay kit. The mixture was then incubated at 37 °C for 30 min. After incubation, the absorbance was measured using a microplate reader (Infinite® 200 Pro, TECAN, Switzerland) at 450 nm. SOD activity was calculated based on the obtained absorbance values and normalized to the bacterial cell numbers [1].

Determination of catalase (CAT) activity

CAT activity was measured according to the manufacturer's instructions, and the procedure was as follows: 10 μl of the supernatant was mixed in 190 μl of working solution, and absorption was measured a microplate reader (Infinite® 200 Pro, TECAN, Switzerland) at 240 nm from starting to 1 min. CAT activity was measured by observing a decrease in the absorbance at 240 nm in time, equivalent to the H_2O_2 degradation by an active enzyme and normalized to the bacterial cell numbers [2].

Determination of malondialdehyde (MDA) content

MDA content was determined according to the manufacturer's instructions, and the procedure was as follows: 100 μL of the supernatant was mixed with 300 μL working solution (volume ratio1:4) and incubated at 100 °C for 60 min. The absorbance of the mixture were then measured using a microplate reader (Infinite® 200 Pro, TECAN, Switzerland) at 600 and 532 nm. MDA content was calculated based on the obtained absorbance values and normalized to the bacterial cell numbers [3].

References:

[1] Hou Z, Zhao L, Wang Y, et al. Purification and characterization of superoxide dismutases from sea buckthorn and chestnut rose. Journal of Food Science. 2019, 84(4): 746–753.

[2] Banerjee, G, Pandey, S, Ray, A.K. et al. Bioremediation of heavy metals by a novel bacterial strain enterobacter cloacae and its antioxidant enzyme Activity, flocculant production, and protein expression in presence of lead, cadmium, and nickel. Water Air Soil Pollution. 2015, 226: 91.

[3] Zhang WX, Tang Y, Han Y, et al. Immunotoxicity of pentachlorophenol to a marine bivalve species and potential toxification mechanisms underpinning. Journal of Hazardous Materials. 2022, 439: 129681.

Table S1. Tail DNA% and olive tail moment obtained from comet assay and the corresponding classification of damage type.

F-53B (mg/L)	Tai DNA (%)	Olive Tai Moment (μm)	Degree of damage
0	1.10 ± 1.98	0.11 ± 0.18	No
0.1	1.31 ± 2.12	0.20 ± 0.52	No
10	16.06 ± 14.69	3.94 ± 4.04	low
50	35.14 ± 18.13	7.83 ± 5.04	Moderate

Note: the relationship between Tail DNA% and the degree of damage is <5%, no damage; 5%~20%, mild damage; 20%~40%, moderate damage; 40%~95%, high damage; >95%, severe damage.
